# Processing of expressions by individuals with autistic traits: Empathy deficit or sensory hyper-reactivity?

**DOI:** 10.1371/journal.pone.0254207

**Published:** 2021-07-09

**Authors:** Chunyan Meng, Chao Huo, Hongxin Ge, Zuoshan Li, Yuanyan Hu, Jing Meng

**Affiliations:** 1 Key Laboratory of Applied Psychology, Chongqing Normal University, Chongqing, China; 2 Laboratory of Emotion and Mental Health, Chongqing University of Arts and Sciences, Chongqing, China; 3 Nanchong Vocational College of Science and Technology, Nanchong, China; SWPS University of Social Sciences and Humanities, POLAND

## Abstract

Individuals with autistic traits display impaired social interaction and communication in everyday life, but the underlying cognitive neural mechanisms remain very unclear and still remain controversial. The mind-blindness hypothesis suggests that social difficulties in individuals with autistic traits are caused by empathy impairment in individuals; however, the intense world theory suggests that these social difficulties are caused by sensory hyper-reactivity and sensory overload, rather than empathy impairment. To further test these two theories, this study investigated event-related potentials (ERPs) to explore the cognitive neural processing of repetitive expressions in individuals with autistic traits. This study employed the Mandarin version of the autism-spectrum quotient (AQ) to assess autistic traits in 2,502 healthy adults. Two subset groups were used, e.g., the participants of a high-AQ group were randomly selected among the 10% of individuals with the highest AQ scores; similarly, the participants in the low-AQ group were randomly selected from the 10% of participants with the lowest AQ scores. In an experiment, three different facial expressions (positive, neutral, or negative) of the same person were presented successively and pseudo-randomly in each trial. Participants needed to define the expression of the face that was presented last. The results showed that compared with the low-AQ group, the high-AQ group exhibited higher P1 amplitudes induced by the second and third presented expressions, as well as higher P3 amplitudes induced by the third presented negative expressions. This indicates that individuals with autistic traits may experience overly strong perception, attention, and cognitive evaluation to repetitive expressions, particularly negative expressions. This result supports the intense world theory more strongly than the mind-blindness hypothesis.

## Introduction

Autism spectrum disorder (ASD) is a neurodevelopmental disorder. Its main features include atypical social interactions and communication patterns, limited interests, as well as rigid and repetitive behaviors [1]. The Diagnostic and Statistical Manual of Mental Disorders (DSM-5) lists “hyper- or hypo- reactivity to sensory input or unusual interests in sensory aspects of environment” as a type of restricted and repetitive behavior. Furthermore, atypical perception has also been defined as a typical feature of ASD [1].

Autistic traits represent a group of major symptoms that are associated with ASD and are continuously distributed throughout the population [2]. This continuous distribution of autistic traits indicates the existence of a quantitative difference in the extent to which individuals display autistic traits, and individuals diagnosed with ASD have stronger autistic traits [3, 4]. The autism-spectrum quotient (AQ) [5] is widely used to measure the level of autistic traits in individuals. With this questionnaire, typically developing people with high AQ scores, who do not fully match the ASD clinical diagnostic criteria, can be identified as individuals with autistic traits [6–8]. It has been reported that individuals with autistic traits have genetic and biological features similar to those of ASD individuals [9, 10]. They show a certain degree of deficit in their social interactions and empathy in their everyday life [11, 12], and exhibit altered sensory processing [13].

Considering the apparent social interaction and communication impairments of ASD individuals, the mind-blindness hypothesis suggests that these are caused by empathy impairment in individuals [14]. Empathy has been defined as the drive of an individual to identify and respond appropriately to emotions and mental states of others, which includes the ability to recognize and understand the emotions of others [15]. The mind-blindness hypothesis holds that empathy impairment causes ASD individuals to experience difficulty in understanding the feelings, thoughts, and beliefs of others, thus causing them to exhibit atypical social interaction patterns [14]. Supporting this hypothesis, studies have shown that ASD individuals have a pronounced difficulty to understand the expressions of others through the eye area [16]. Thus, the accuracy rate of ASD individuals for identifying disgust and sadness is lower than that of typical individuals [17]. It has also been found that individuals with autistic traits are insensitive to emotional faces of fear [18]. Furthermore, daily observation and field experiments showed that ASD individuals have less empathic responses than typically developing individuals [19, 20]. Individuals with high AQ have also been found to have a lower ability to recognize the facial expressions of others, and they make more mistakes when asked to identify facial expressions than individuals with low AQ [6, 21].

The intense world theory introduced a contrasting view to the mind-blindness hypothesis, by suggesting that ASD individuals are not unable to empathize with others, but rather overreact to the emotions of others [22]. The intense world theory proposes that ASD individuals apply overly strong perception, attention, and emotional responses to sensory stimuli, which thus results in the processing of a large volume of sensory information. This causes ASD individuals to experience fear and anxiety, in response to which they may avoid normal social and emotional communication [22]. Therefore, the empathy impairment displayed by ASD individuals in their everyday lives may be caused by sensory hyper-reactivity. When stimuli were presented repetitively, ASD individuals showed a stronger response to sensory stimuli than control individuals [23]. In this study, both visual (checkerboard) and auditory (white noise) modality stimuli were presented. The results showed that when these stimuli were presented only once, no difference was found in the response between ASD individuals and a control group; however, when the stimuli were presented repetitively, the activation in the auditory cortex region of ASD individuals was stronger than that of control individuals [23]. Our recent study [24] used event-related potentials (ERPs) to measure the empathic neural responses that were induced by the repetition of three identical audio recordings (human voices, S1_S2_S3) in both high-AQ and low-AQ groups. P2 amplitudes of the second repetition were significantly higher for high-AQ groups rather than for low-AQ groups. These results suggest that ASD individuals respond strongly to repetitive auditory stimuli (both pure stimuli and social stimuli). Moreover, they show a general sensory processing sensitivity that supports the intense world theory. However, for visual stimuli (i.e., checkerboard), no differences were found between ASD individuals and control individuals with regard to the activation of the visual cortex region [23]. A previous study [25] found significantly reduced Amygdala habituation to faces (i.e., a change in response over time) but not to houses in ASD individuals. Another study also found that ASD individuals had a reduced ability to maintain habituation in the amygdala across repeated sensory stimulation [26]. Combined with the results of visual stimulation, it can be assumed that ASD individuals show a specific over sensitivity to emotional processing of social stimuli, which contrasts with the mind-blindness hypothesis [14]. Therefore, the present study uses emotional expression stimuli in the paradigm [23] to test whether individuals with autistic traits overreact to emotional stimuli at the visual level, which would support the intense world theory.

Thus, the mind-blindness hypothesis [14] would argue that the recognition of emotions is specifically impaired in autistic individuals, whereas the intense world theory [22] would suggest that the recognition of emotions is not specifically impaired in autistic individuals, even reacts hyper-reactivity. Therefore, in the conducted experiment, participants were asked to judge the expressions of presented faces to test whether their ability of emotion recognition is specifically impaired.

According to mind-blindness hypothesis, ASD individuals have an amygdala deficit [27, 28]; thus, no matter how many times emotional faces are presented to ASD individuals, their neural response may be weaker than in typically developing individuals. However, the intense world theory holds that ASD individuals have a hyper-reactive amygdala [29], and considerable evidence showed that ASD individuals indeed have such higher amygdala activation during the processing of emotional faces compared with typical individuals [30, 31]. Reduced habituation (i.e., a change in response over time) was also found in the amygdala of ASD individuals [25]. Habituation is pervasive in sensory systems; it refers to changes in neural and behavioral responses that accompany prolonged exposure to an adapting stimulus with repeated features [24, 32]. This may reflect that ASD individuals have a stronger neural response with increasing appearance of emotional faces. Therefore, the revised paradigm of repetitive stimulus [23, 25] was used in the current study to investigate the cognitive and neural responses (e.g., perception, attention) of individuals with autistic traits to others’ repetitive expressions. The results were used to test whether one of these theories could predict the responses of individuals. The stimuli were arranged so that three different facial expressions (i.e., positive, neutral, and negative) of the same person were presented both successively and pseudo-randomly, and participants were asked to judge the expressions of the face that was presented last.

An electroencephalogram (EEG) can provide information relevant to neural activities, and analysis of ERPs is well-suited for assessing attention underlying processing of facial expressions [33]. The P1 component of an ERP reflects the distribution of attention for the processing of sensors and faces [34, 35]. N170 is presumed to reflect the perceptual processing of information obtained from faces [36, 37]. P3 components over the posterior parietal area have been linked to cognitive evaluation and sustained attentional processing of emotional stimuli [38, 39]. The late positive component (LPC) is a neurophysiological indicator of emotional responses [40], and can be used to identify the motivational attention of emotional stimuli [41, 42]. Thus, this study used ERP technology to investigate the responses of individuals with autistic traits to others’ repetitive expressions.

Two hypotheses are proposed in this study. Hypothesis 1: based on the mind-blindness hypothesis [14], the ERP amplitudes (P1 or N170) in response to the first expression (without the effect of stimuli repetition) in the high-AQ group should be smaller than that of the low-AQ group. No significant change was observed in the ERP amplitudes (P1 or N170) as the number of faces increased in the high-AQ group. However, based on the intense world theory [22], in the early stages of emotional processing, ERP amplitudes (P1 or N170) in response to the second and third expressions (with the effect of stimuli repetition) in the high-AQ group should be higher than those of the low-AQ group. Furthermore, the ERP amplitudes (P1 or N170) in the high-AQ group would increase with the repeated presentation of expressions.

Additionally, previous studies suggested that providing rich social stimulation can enhance the emotion recognition and reactivities of ASD individuals [14, 43]. When emotional face appears repeatedly, the recognition of expressions and neural activity of ASD individuals may improve. Therefore, Hypothesis 2 is proposed: based on the mind-blindness hypothesis [14], with increasing number of face presentations, the ability to recognize emotional faces would enhance in the high-AQ group, and their ability to correctly identify facial emotions matches that of the low-AQ group. Specifically, there will be no significant difference in the accuracy and response rate of expression recognition between the high-AQ group and the low-AQ group. When three different emotional faces of the same person are presented in order and pseudo-randomly, the amplitude (P3 or LPC) of the ERP components to those expressions in the high-AQ group will increase. However, the intense world theory [22] holds that the recognition of expressions by individuals with ASD may be reduced because of emotional information overload. Recent studies have also shown that sensory abnormalities can affect the responses of ASD individuals to the expressions of others [44], as well as their social and emotional communication [45]. Therefore, based on the intense world theory [22], at the late-emotional processing stage, compared with the low-AQ group, the high-AQ group will have a lower rate of correct answers and a longer response time in expression recognition with repetition, which would be caused by emotional information overload. The amplitude (P3 or LPC) to repetitive expressions in the high-AQ group will decrease (from the first expression to the second and third expressions). Moreover, when those facial expressions are presented for the third time, compared with the low-AQ group, the high-AQ group will have lower amplitude (P3 or LPC) of expressions because of emotional information overload.

## Materials and methods

### Participants

A total of 2,502 university students at the Chongqing Normal University, China, aged 18–26 (M = 21.07 years, SD = 2.11 years) were recruited to complete the Mandarin version [46] of the AQ questionnaire [5]. Their responses were used to estimate their autistic traits. Thirty participants (15 females) were randomly selected from the 10% of students with the highest AQ scores and were identified as the high-AQ group. A further 30 participants (15 females) were randomly selected from the 10% of students with the lowest AQ scores, and were identified as the low-AQ group [47, 48]. Their ages and AQ scores are summarized in Table 1.

**Table 1:** Ages and AQ scores of high-AQ group and low-AQ group participants in the study.

	Age					AQ Score				
	Min	Max	*M ± SD*	*t*	*p*	Min	Max	*M ± SD*	*t*	*p*
High-AQ group	18	25	20.77 ± 1.54	1.101	0.276	27	33	29.07 ± 1.74	35.136	< 0.001
Low-AQ group	18	26	21.37 ± 2.55			9	15	12.77 ± 1.85		

Note. AQ = Autism Spectrum Quotient. *p*-values and *t* values were obtained from independent samples *t*-tests performed on ages and AQ scores between high-AQ and low-AQ group participants.

In accordance with the Declaration of Helsinki, all participants provided free and informed consent before the experiment and all procedures were approved by the research ethics committee of Chongqing Normal University. The procedures were performed in accordance with current ethical guidelines and regulations issued by this committee.

### Materials

A total of 24 pictures of the faces of eight models with three different expressions (positive/happy, neutral, negative/sad) were selected from the Chinese facial affective picture system (CFAPS) [49]. Before the experiment, 40 undergraduate students (20 females) who did not participate in the experiment were asked to score the valence (1 = very unhappy, 9 = very happy) and arousal (1 = extremely peaceful, 9 = extremely excited) of the emotional pictures using 9-point Likert scales. Significant differences were found in emotional valence among the three types of expressions (*F*_*1*, *38*_ = 94.31, *p* < 0.001, $\eta_{\text{p}}^{2}=0.71$), with participants accurately judging positive, neutral, and negative expressions (positive: 6.8 ± 0.60, neutral: 4.42 ± 0.52, negative: 3.06 ± 0.56). No significant differences were found for arousal (*F*_*1*, *38*_ = 3.05, *p* = 0.069, $\eta_{\text{p}}^{2}=0.07$; positive: 5.59 ± 0.41, neutral: 4.91 ± 0.51, negative: 5.46 ± 0.90).

### Procedure

Participants were seated in a quiet room with an ambient temperature of about 20°C, with their faces about 80 cm away from a computer-controlled monitor (1980 × 1080 pixels). The image size was 13.5 cm × 11.5 cm (width × height) and a viewing angle of about 9.6° × 8.2° was applied. The test items were presented in a pseudo-random order. Stimulus presentation was controlled using the E-Prime (3.0) program (Psychology Software Tools, Pittsburgh, PA, USA).

**Fig 1:**
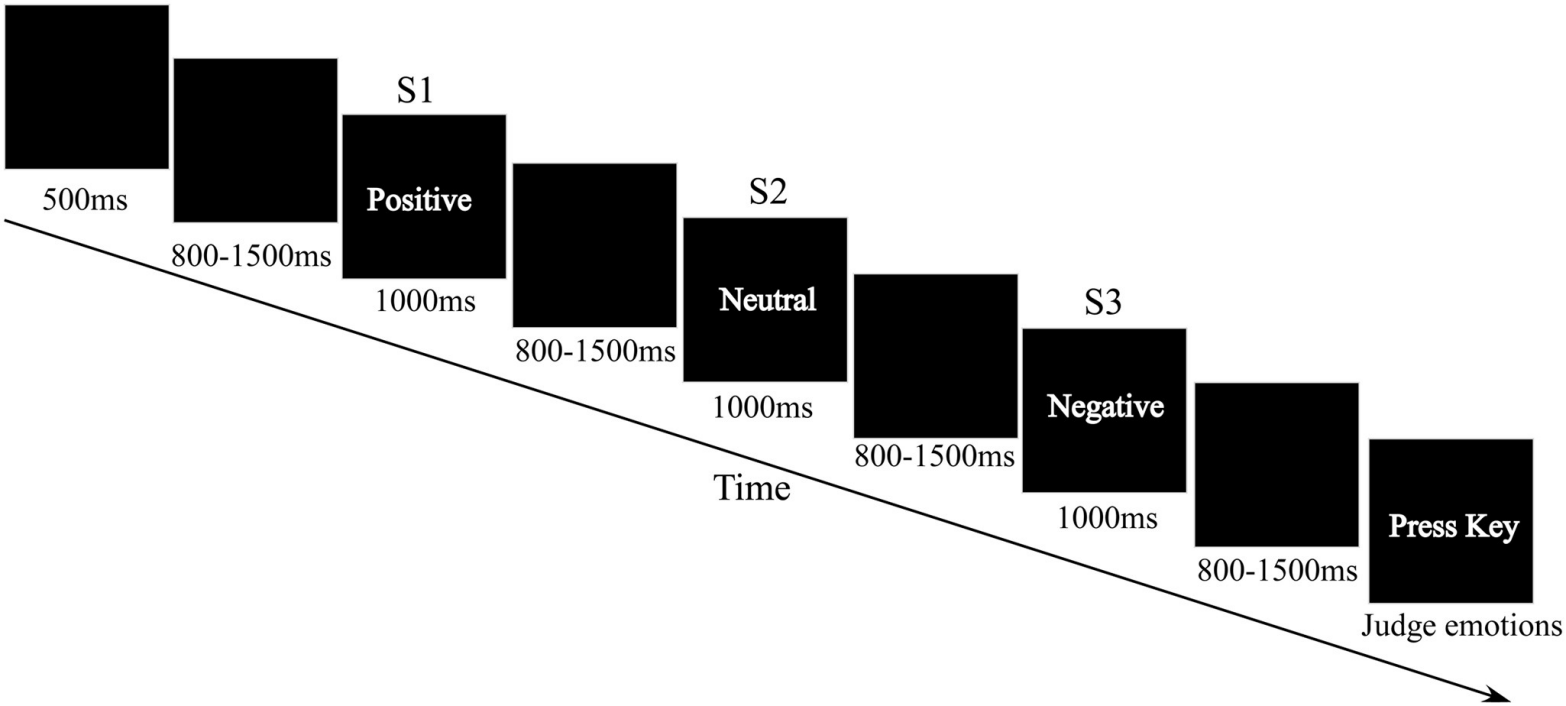
Flowchart describing the experimental design of experiments. All pictures in the experiment were selected from the Chinese facial affective picture system (CFAPS) (Bai et al., 2005). The words “Positive”, “Neutral”, and “Negative” in Fig 1 represent pictures with faces that show positive, neutral, or negative emotional expressions, respectively. During each trial, sets of pictures were delivered on a computer screen. Each set consisted of three expressions from one model (S1-S2-S3, a triplet), delivered at a random ISI of 800–1,500 ms. The order in which expressions (positive, neutral, and negative) were presented randomly. Participants were instructed to respond as accurately and quickly as possible by pressing a key to identify the last presented expression (S3) as soon as the words “press key” appeared on the screen.

Similar to previous studies [50, 51], in each trial (of 70% of the total trials), sets of three expressions from one model (S1-S2-S3, a triplet) were presented, delivered at a random inter-stimulus interval (ISI) of 800–1,500 ms (Fig 1). Additionally, as the emotion recognition task may drive the attention of participants, which may result in participants paying more attention to the S3 expressions while ignoring S1 and S2 expressions, in 15% of the total trials, participants were asked to judge the emotions after the presentation of the first expressions (only S1 were presented in these trials). In another 15% of the total trials, the participants were asked to judge the emotions after the expressions had been presented twice (S1 and S2 were presented in these trials). Only the data from trials with S1-S2-S3 triplet were analyzed. All trials used a pseudo-random arrangement.

An example trial is displayed in Fig 1. At the start of the trial, a fixation cross was presented on a black screen for a duration of 500 ms; then, a black screen was presented for a duration of 800–1500 ms (the length was random within this interval); and then, three expressions (positive, neutral, or negative) from a triplet were pseudo-randomly presented for 1000 ms, with an ISI of 800–1,500 ms. Following the stimulus triplet, participants were instructed to respond as accurately and quickly as possible to a text signal (“press key”, which appeared 500 ms after S3) by pressing a specific key (“1”, “2”, or “3”) on the keyboard to define whether the last presented expression was positive, neutral, or negative, respectively. The text signal disappeared from the screen as soon as the participants pressed the key. The key-pressing was counterbalanced across participants to control for possible order effects. The inter-trial interval was 3–4 s. Thus, the experiment consisted of three blocks and each block included 107 trials. Prior to the experiment, each participant conducted a training session of about nine trails to familiarize with the procedure.

### Electroencephalography recording

EEG data were recorded from 64 scalp sites using tin electrodes located according to the international 10–20 system that was mounted on an actiCHamp system (Brain Vision LLC, Morrisville, NC, USA). The electrode at the right mastoid was used as recording reference, while that on the medial frontal aspect was used as ground electrode. EEG activities were amplified with a 0.01–100 Hz bandpass and were continuously sampled at 1000 Hz. All electrode impedances remained below 5 kΩ.

### Data analysis

EEG data were pre-processed and analyzed via MATLAB R2016a (MathWorks, USA) and the EEGLAB toolbox [52]. EEG signals were passed through an off-line 0.1–40 Hz band-pass filter. Time windows of 200 ms before and 1,000 ms after the onset of stimuli were extracted from the continuous EEG and the extracted window was baseline-corrected by the 200 ms time interval prior to stimuli onset. EEG epochs with amplitudes exceeding ± 80 μV at any electrode were excluded from further analyses. EEG epochs were also visually inspected, and trials that were contaminated by gross movements were excluded. Electro-oculogram (EOG) artifacts were corrected via the independent component analysis (ICA) algorithm [53]. These epochs constituted 1.50 ± 2.32% of the total number of epochs.

According to previous studies and the location of topographical maps of maximum averaged ERP activity [34, 54–56], the sites of the analysis electrode were determined. The electrode sites of P1 were O1, Oz, O2, PO3, POz, and PO4, and the latency interval was 130–140 ms. The electrode sites of P3 and LPC components were CP1, CPZ, CP2, P1, Pz, and P2, and the latency intervals of P3 and LPC were 300–340 ms and 400–600 ms, respectively. The electrode sites of N170 components were PO7 and PO8, and the latency interval of N170 was 160–180 ms.

The recorded accuracies (ACCs) and response times (RTs) for S3 were compared via two-way repeated-measures analyses of variance (ANOVA), using one within-participant factor of “expression” (positive, neutral, and negative), and one between-participants factor of “group” (high-AQ group vs. low-AQ group). The average amplitudes of ERP components were compared via three-way repeated-measures ANOVA, using two within-participant factors of “sequence” (S1, S2, and S3), and “expression” (positive, neutral, and negative), as well as the between-participants factor of “group” (high-AQ group vs. low-AQ group). The degrees of freedom for F-ratios were corrected according to the Greenhouse-Geisser method [57]. If significant, post hoc analysis with the factor “group” was performed for each condition.

## Results

### Behavioral results

The descriptive statistical results of the RTs and the ACCs of both groups of participants for different expressions are shown in Table 2. The results of the statistical analysis of behavioral data are shown in Table 3.

**Table 2:** RTs and ACCs of both groups in the study (M ± SD).

	High-AQ group			Low-AQ group		
	Positive	Neutral	Negative	Positive	Neutral	Negative
**RTs (ms)**	566.92±201.39	583.19±198.09	613.68±247.18	522.81±169.15	545.44±157.83	538.73±176.01
**ACCs (%)**	93.65±7.33	88.92±8.56	81.24±15.82	96.57± 5.18	91.00±10.82	88.84±16.01

**Table 3:** Summary of statistical analysis results for the behavioral data.

	RTs			ACCs		
	*F*	*p*	*$\eta_{\text{p}}^{2}$*	*F*	*p*	*$\eta_{\text{p}}^{2}$*
**Expression**	**4.48**	**0.013**	**0.07**	**15.26**	**< 0.001**	**0.21**
**Group**	1.16	0.286	0.02	**4.17**	**0.046**	**0.07**
**Expression × Group**	1.77	0.174	0.03	0.65	0.423	0.01

Note: df: (1,58), The significant comparisons (*p* < 0.05) were shown in boldface.

For RTs, the main effect of “expression” was significant (*F*_*1*, *58*_ = 4.48, *p* = 0.013, $\eta_{\text{p}}^{2}=0.07$). The RTs for positive expressions (544.87 ± 185.73 ms) were significantly shorter than the RTs for neutral expressions (564.32 ± 178.59 ms; *p* = 0.032) and negative expressions (576.20 ± 216.07 ms; *p* = 0.009). No significant difference (*p* = 0.287) was found between the RTs for neutral expressions (564.32 ± 178.59 ms) and negative expressions (576.20 ± 216.07 ms).

For ACCs, the main effect of “expression” was significant (*F*_*1*, *58*_ = 15.26, *p* < 0.001, $\eta_{\text{p}}^{2}=0.21$). The ACCs for positive expressions (95.11 ± 6.46%) were significantly higher than those for neutral expressions (89.95 ± 9.73%; *p* < 0.001) and negative expressions (85.04 ± 16.24%; *p* < 0.001). The ACCs for negative expressions (85.04 ± 16.24%) were significantly lower than those for neutral expressions (89.95 ± 9.73%; *p* = 0.034). The main effect of “group” was significant (*F*_*1*, *58*_ = 4.17, *p* = 0.046, $\eta_{\text{p}}^{2}=0.07$), the ACCs of the high-AQ group (87.93 ± 8.34%) were significantly lower than that of the low-AQ group (92.14 ± 7.54%). No other main effect or interaction effect was significant (all *p*-values > 0.05).

### ERP results

#### ERP amplitudes

**Fig 2:**
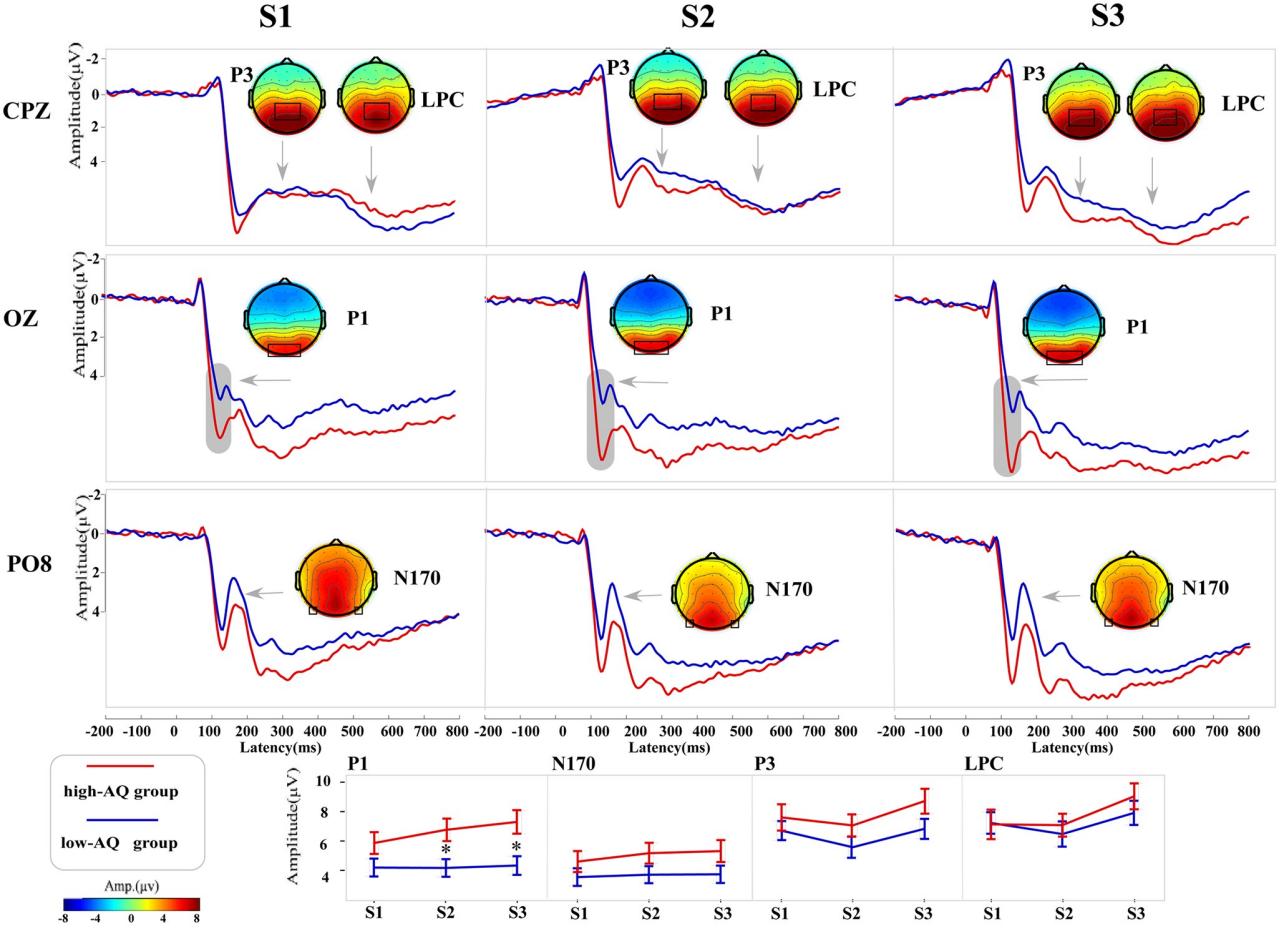
ERP waveforms and topographic maps (top panel), as well as line charts (bottom panel) of high-AQ (red line) and low-AQ (blue line) groups. S1, S2, and S3 represent the first, second, and third presented facial expressions, respectively. Electrodes used to estimate the mean ERP amplitudes were marked using black squares on their respective topographic distributions. Data in the line charts are expressed as Mean ± SEM. *: *p* < 0.05.

**Fig 3:**
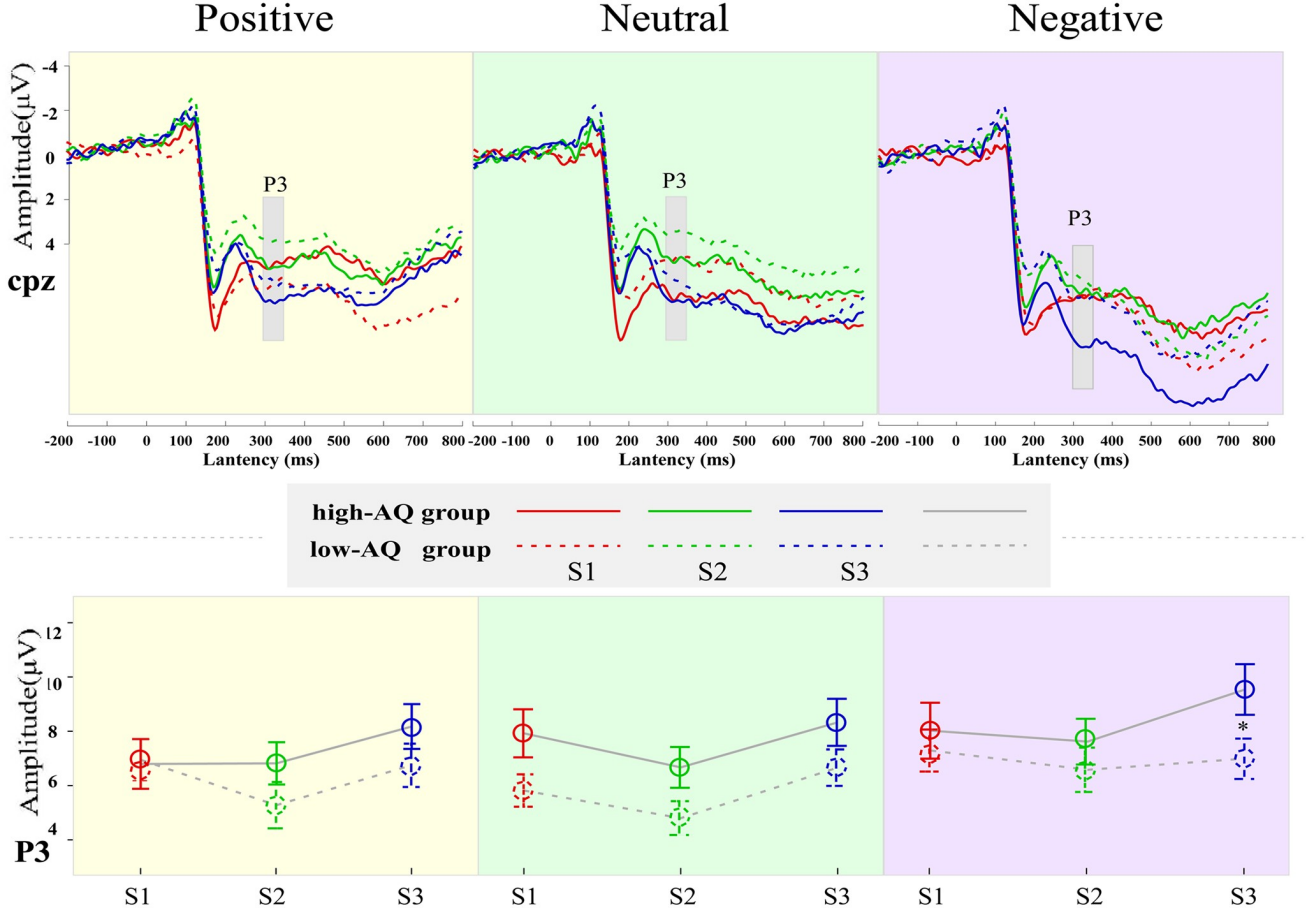
ERP waveforms and line charts of high-AQ and low-AQ groups. ERP waveforms (top panel) and line charts (bottom panel) of high-AQ (solid line) and low-AQ (dotted line) groups for positive (yellow background color), neutral (green background color), and negative (pink background color) facial expressions. S1 (red line), S2 (green line), and S3 (blue line) represent the first, second, and third presented facial expressions, respectively. Line charts represent P3 amplitudes. Data in the line charts are expressed as Mean ± SEM. *: *p* < 0.05.

The analysis results for the variance of ERP data are shown in Table 4. Waveforms and topographic maps under different conditions are shown in Figs 2 and 3.

**Table 4:** Summary of statistical analysis results of ERP data.

	P1			N170			P3			LPC		
	*F*	*p*	$\eta_{\text{p}}^{2}$	*F*	*p*	$\eta_{\text{p}}^{2}$	*F*	*p*	$\eta_{\text{p}}^{2}$	*F*	*p*	$\eta_{\text{p}}^{2}$
**Expression**	**5.13**	**0.007**	**0.08**	**9.33**	**<0.001**	**0.17**	**10.93**	**<0.001**	**0.16**	**28.27**	**<0.001**	**0.33**
**Sequence**	**10.14**	**<0.001**	**0.15**	**3.53**	**0.035**	**0.06**	**7.17**	**0.003**	**0.11**	**7.00**	**0.004**	**0.11**
**Group**	**6.25**	**0.015**	**0.10**	2.25	0.139	0.04	2.09	0.154	0.04	0.26	0.614	0.01
**Expression × Group**	**6.50**	**0.002**	**0.10**	**6.89**	**0.001**	**0.11**	2.12	0.125	0.04	1.35	0.264	0.02
**Sequence × Group**	**7.25**	**0.002**	**0.11**	1.15	0.317	0.02	0.84	0.406	0.01	0.84	0.400	0.01
**Expression × Sequence**	0.86	0.481	0.02	0.77	0.522	0.01	0.60	0.638	0.01	1.04	0.382	0.02
**Expression × Sequence × Group**	1.64	0.173	0.03	0.30	0.842	0.01	**2.96**	**0.028**	**0.05**	**4.86**	**0.001**	**0.08**

Note: df: (1, 58), The significant comparisons (*p* < 0.05) were shown in boldface.

#### P1

The main effect of “expression” was significant (*F*_*2*, *57*_ = 5.13, *p* = 0.007, $\eta_{\text{p}}^{2}=0.08$). The average amplitudes of negative expressions (5.60 ± 4.17 μV; *p* = 0.006) and neutral expressions (5.51 ± 3.84 μV; *p* = 0.028) were significantly higher than the average amplitudes of positive expressions (5.20 ± 3.80 μV). The average amplitudes of negative expressions (5.60 ± 4.17 μV) and neutral expressions (5.51 ± 3.84 μV) did not differ significantly (*p* = 0.444). The main effect of “sequence” was significant (*F*_*2*, *57*_ = 10.14, *p* < 0.001, $\eta_{\text{p}}^{2}=0.15$). The average amplitudes of the third presented expressions (S3) (5.81 ± 4.20 μV) were significantly higher than those of the first presented expressions (S1) (5.03 ± 3.77 μV; *p* < 0.001) and the second presented expressions (S2) (5.46 ± 3.96 μV; *p* = 0.017). The main effect of “group” was significant (*F*_*1*, *58*_ = 6.25, *p* = 0.015, $\eta_{\text{p}}^{2}=0.10$). The interaction between “expression” and “group” was significant (*F*_*1*, *58*_ = 6.50, *p* = 0.002, $\eta_{\text{p}}^{2}=0.10$). As indicated by simple effect analysis, the average amplitudes of the high-AQ group (6.85 ± 4.15 μV) for neutral expressions were significantly higher than those of the low-AQ group (6.17 ± 3.02 μV) (*F*_*1*, *58*_ = 8.12, *p* = 0.006, $\eta_{\text{p}}^{2}=0.12$); moreover, the average amplitudes of the high-AQ group (6.94 ± 4.39 μV) for negative expressions were significantly higher than those of the low-AQ group (4.25 ± 3.5 μV) (*F*_*1*, *58*_ = 6.88, *p* = 0.011, $\eta_{\text{p}}^{2}=0.11$). The interaction between “sequence” and "group” was significant (*F*_*2*, *57*_ = 7.25, *p* = 0.002, $\eta_{\text{p}}^{2}=0.11$). As shown by the simple effect analysis, the P1 amplitudes of high-AQ group were significantly higher than those of the low-AQ group in response to S2 (*F*_*1*, *58*_ = 7.08, *p* = 0.010, $\eta_{\text{p}}^{2}=0.11$) and S3 (*F*_*1*, *58*_ = 8.41, *p* = 0.005, $\eta_{\text{p}}^{2}=0.01$) expressions; however, no significant difference to S1 expression was found between groups (*F*_*1*, *58*_ = 3.04, *p* = 0.086, $\eta_{\text{p}}^{2}=0.05$).

#### N170

The main effect of “expression” was significant (*F*_*2*, *57*_ = 9.33, *p* < 0.001, $\eta_{\text{p}}^{2}=0.17$). The N170 amplitudes of negative expressions (4.57 ± 3.65 μV) were significantly higher than those of neutral expressions (4.26 ± 3.49 μV; *p* = 0.002) and positive expressions (4.11 ± 3.65 μV; *p* < 0.001). The N170 amplitudes of neutral expressions (4.26 ± 3.49 μV) and positive expressions (4.11 ± 3.65 μV) did not differ significantly (*p* = 0.192). The main effect of “sequence” was significant (*F*_*2*, *57*_ = 3.53, *p* = 0.035, $\eta_{\text{p}}^{2}=0.06$), and the amplitudes of S3 expressions (4.49 ± 3.74 μV) were significantly higher than those of S1 expressions (4.04 ± 3.62 μV; *p* = 0.023). No significant difference was found between the N170 amplitudes of S2 (4.41 ± 3.59 μV) and S3 (4.49 ± 3.74 μV) expressions (*p* = 0.573). The interaction between “expression” and “group” was significant (*F*_*2*, *57*_ = 6.89, *p* = 0.001, $\eta_{\text{p}}^{2}=0.11$). As shown by the simple effect analysis, the main effect of “expression” was significant in the high-AQ group (*F*_*2*, *57*_ = 12.57, *p* < 0.001, $\eta_{\text{p}}^{2}=0.31$). The N170 amplitudes of negative expressions (5.39 ± 3.93 μV) were significantly higher than those of both neutral (5.05 ± 3.88 μV; *p* < 0.001) and positive (4.56 ± 3.93 μV; *p* = 0.016) expressions, and the amplitudes of neutral expressions (5.05 ± 3.88 μV) were significantly higher than those of positive expressions (4.56 ± 3.93 μV; *p* = 0.004). However, no main effect of “expression” was found in the low-AQ group (*F*_*2*, *57*_ = 2.14, *p* = 0.128, $\eta_{\text{p}}^{2}=0.07$).

#### P3

The main effect of “expression” was significant (*F*_*2*, *57*_ = 10.93, *p* < 0.001, $\eta_{\text{p}}^{2}=0.16$). The P3 amplitudes induced by negative expressions (7.66 ± 4.18 μV) was significantly higher than that induced by both positive expressions (6.79 ± 3.98 μV; *p* < 0.001) and neutral expressions (6.68 ± 3.74 μV; *p* < 0.001). The main effect of “sequence” (*F*_*2*, *57*_ = 7.17, *p* = 0.003, $\eta_{\text{p}}^{2}=0.11$) was significant. The P3 amplitudes of S3 expressions (7.73 ± 4.27 μV) were significantly higher than those of S2 expressions (6.28 ± 4.06 μV; *p* < 0.001). The P3 amplitudes of S1 expressions (7.12 ± 4.27 μV) was significantly higher than that of S2 expressions (6.28 ± 4.06 μV; *p* = 0.021). The interaction among “expression”, “sequence”, and “group” was significant (*F*_*4*, *55*_ = 2.96, *p* = 0.028, $\eta_{\text{p}}^{2}=0.05$). As found by simple effect analysis, with regard to the negative expressions, the P3 amplitudes of the high-AQ group were significantly higher than that of the low-AQ group in response to S3 expressions (*F*_*1*, *58*_ = 4.56, *p* = 0.037, $\eta_{\text{p}}^{2}=0.07$). However, no significant difference was found to S1 (*F*_*1*, *58*_ = 0.32, *p* = 0.573, $\eta_{\text{p}}^{2}=0.006$) and S2 (*F*_*1*, *58*_ = 0.78, *p* = 0.381, $\eta_{\text{p}}^{2}=0.01$) expressions. Furthermore, in the high-AQ group, the main effect of the sequence of negative expression was significant (*F*_*2*, *57*_ = 8.31, *p* = 0.001, $\eta_{\text{p}}^{2}=0.23$). The P3 amplitudes of the negative S3 expressions (9.52 ± 5.11 μV) were significantly higher than those of the negative S2 expressions (7.60 ± 4,61 μV; *p* < 0.001), but no significant difference was found in the low-AQ group. No significant difference between groups was found in response to positive and neutral expressions (all *p*-values > 0.05).

#### LPC

The main effect of the “expression” was significant (*F*_*2*, *57*_ = 28.27, *p* < 0.001, $\eta_{\text{p}}^{2}=0.33$). The LPC amplitudes induced by negative expressions (8.71 ± 4.88 μV) were significantly higher than those induced by both positive expressions (6.56 ± 4.19 μV; *p* < 0.001) and neutral expressions (6.93 ± 3.85 μV; *p* < 0.001). The LPC amplitudes induced by positive expressions (6.56 ± 4.19 μV) and neutral expressions (6.93 ± 3.85 μV) did not differ significantly (*p* = 0.222). The main effect of “sequence” (*F*_*2*, *57*_ = 7.00, *p* = 0.004, $\eta_{\text{p}}^{2}=0.11$) was significant. The LPC amplitudes of S3 (8.40 ± 4.65 μV) were significantly higher than those of S1 (7.11 ± 4.75 μV; *p* = 0.033) and S2 (6.70 ± 4.40 μV; *p* < 0.001) expressions. No significant difference was found between the LPC amplitudes of S1 (7.11 ± 4.75 μV) and S2 (6.70 ± 4.40 μV) (*p* = 0.392) expressions. The interaction among “expression”, “sequence”, and “group” was significant (*F*_*4*, *55*_ = 4.96, *p* = 0.001, $\eta_{\text{p}}^{2}=0.08$). As identified by the simple effect analysis, in the high-AQ group, the main effect of negative expression “sequence” was significant (*F*_*2*, *57*_ = 10.76, *p* < 0.001, $\eta_{\text{p}}^{2}=0.27$). The LPC amplitudes of the negative S3 expressions (10.71 ± 5.81 μV) were significantly higher than those of both the negative S1 expressions (8.08 ± 6.70 μV; *p* = 0.024) and negative S2 expressions (7.97 ± 4.96 μV; *p* < 0.001). No other main effect or interaction effect was significant (all *p*-values > 0.05).

#### ERPs latency

As delayed responses have also frequently been found in P1 or N170 in ASD individuals when processing facial expressions, the ERP latencies of P1 and N170 were examined. No significant differences were found between the high-AQ group and the low-AQ group for the latencies of P1 (*F*_*1*, *58*_ = 0.03, *p* = 0.875, $\eta_{\text{p}}^{2}<0.001$) and N170 (*F*_*1*, *58*_ = 3.01, *p* = 0.088, $\eta_{\text{p}}^{2}=0.05$). Furthermore, no significant differences in interactions among “expression”, “sequence”, and “group” were found in P1, N170, and P3 (all p-values > 0.05). Details are shown in S1 Table.

## Discussion

This study investigated the neural processing of expressions (S1-S2-S3) by individuals with autistic traits, using ERP technology. The results showed that compared with the low-AQ group, the high-AQ group had a lower accuracy for recognizing facial expressions, higher P1 amplitudes induced by the second and third presented (S2 and S3) expressions, and higher P3 amplitudes induced by the third presented (S3) negative expressions. This indicates that compared with the low-AQ group, individuals with high-AQ may have an overly strong perception, evaluation, and attention to repetitive expressions, particularly, negative expressions. Together, findings support the intense world theory more strongly than the mind-blindness hypothesis.

The behavioral results show that the RTs to positive expressions were significantly faster compared with neutral and negative expressions; moreover, the ACCs of recognizing positive expressions were significantly higher than the ACCs of neutral expressions and negative expressions, both of which were consistent with the results of previous studies [55]. This may be because positive expressions are more familiar in social life than the other presented expressions, and thus, their presentation facilitates perceptual reaction [55, 58], which may lead to quicker and more accurate processing of positive expressions. The high-AQ group had a much lower ACCs for recognizing facial expressions compared with the low-AQ group. This is consistent with previous studies on individuals with autistic traits [6, 21] and ASD [17, 59]. This indicates that compared with the low-AQ group, individuals in the high-AQ group may have a weaker ability to correctly recognize facial expressions, and may thus, display weaker categorization boundaries [60].

In the present study, the high-AQ group showed higher P1 amplitudes to negative and neutral expressions than the low-AQ group. The P1 component reflects the distribution of attention for sensory and facial processing [34, 35], and its increase in amplitude has been associated with increased cognitive resources to perceive faces holistically [61, 62]. This result indicates that compared with the low-AQ group, individuals with high-AQ may have an increased perception and attention to negative facial expressions. In addition, no significant difference was found in the P1 amplitudes between the high-AQ and the low-AQ groups to S1 expressions, which is comparable to previous studies in which expressions were presented only once [63, 64]. Interestingly, the high-AQ group showed higher P1 amplitudes than the low-AQ group to S2 and S3 expressions. That is, the differences of P1 amplitudes between the high-AQ and low-AQ groups increased with expressions. This indicates that compared with the low-AQ group, individuals with high-AQ may have an overly strong perception for and attention to repetitive stimulation by facial expressions at early ERP responses. This result supports both Hypothesis 1 and the intense world theory.

Hypothesis 2 predicts that in the late-emotional processing stage, the amplitude of the high-AQ group should either increase (mind-blindness hypothesis) or decrease (intense world theory) with increasing number of expression stimuli. However, this study showed that the amplitude of P3 and LPC followed a decreasing trend from the first expressions to the second expressions, but increased with the appearance of the third expressions. This result was partly in line with expectation. This may be related to the top-down attention to facially presented emotions of participants. In the experiment, participants had to judge the expressions of the face after the third presentation, so that they could be more attentive to the emotion of the third expressions. Interestingly, as shown in the present study, relative to the low-AQ group, the P3 amplitudes of the negative S3 expressions were significantly higher than those of the negative S2 expressions, and the P3 amplitudes significantly increased in the negative S3 expressions in the high-AQ group. P3 components over the posterior parietal area have been linked to cognitive evaluation and sustained attentional processing of emotional stimuli [38, 39]. This might contribute to emotional regulation and social understanding [65, 66]. Thus, individuals with high-AQ may apply overly strong cognitive evaluation and attention to repeated negative expressions at late ERP responses. Previous studies in ASD individuals also showed that the orientation of negative expressions by ASD individuals may cause excessively strong emotional responses in them, thus leading to avoidance behavior with regard to such stimuli [67]. Thus, in the present study, individuals with high-AQ may also show increased late cortical responses to repeated negative expressions. This result also supports the intense world theory.

Combining behavioral data and neural data showed that compared with the low-AQ group, the high-AQ group had a lower ACC for recognizing facial expressions, while the high-AQ group had higher P1 amplitudes induced by the second and third presented (S2 and S3) expressions, and higher P3 amplitudes induced by the third presented (S3) negative expressions. This may reflect that the repeated occurrence of facial stimuli affects the recognition of emotional faces in the high-AQ group, which supports the intense world theory. The authors therefore propose that high-AQ individuals have strong perception and attention to facial information, which causes information overload and affects their integration of facial emotional information. At the same time, the intense world theory proposes that once the attention is captured, individuals with ASD may experience difficulty to shift their attention to different features or tasks [22]. Therefore, excessively strong attention to negative emotions in individuals with high-AQ may affect the recognition of positive and neutral faces. On the other hand, in the high-AQ group, the LPC amplitudes of negative S3 expressions were significantly higher compared with those of both the negative S1 expressions and negative S2 expressions. LPC has been used to indicate the motivational attention of emotional stimuli [41, 42], and its increase in amplitude has been associated with stronger avoidance motivation for negative stimuli [39, 68]. This may indicate that at the late stage of emotional processing, individuals with high-AQ may experience stronger motivation to avoid negative stimuli with increasing presentation of facial stimuli. The overly strong perception of negative emotions by high-AQ individuals may cause strong emotional responses, such as excessive fear and anxiety; consequently, they adopt an avoidance strategy to calm the discomfort caused by strong emotional reactions [69]. Combined with these results, high-AQ individuals have a much lower ACC for recognizing facial expressions compared with low-AQ individuals, which supports the intense world theory.

In summary, this study shows that, compared with the low-AQ group, individuals with autistic traits directed an overly strong perception, attention, and cognitive evaluation to repeated expressions (especially negative expressions) in both early and late ERP components, which may affect their behavioral responses in daily life. According to the intense world theory [22], when ASD individuals are processing repeated expressions, a number of regions of the neocortex (e.g., the prefrontal cortex, sensory cortex, and amygdala) are overly active, thus magnifying their sensory experience. This may put them in a state of sensory information overload, and causes the association of excessive fear and anxiety with social stimuli (e.g., facial expressions). As a result, social withdrawal can be triggered [70]. Such overly strong responses may also cause information overload in individuals with autistic traits. The results of this study support the intense world theory [22] more strongly than the mind-blindness hypothesis [14].

## Limitation

Despite possible implications, the limitations of the study should be addressed. This study assumed that the amplitudes (P3 or LPC) to repetitive expressions in the high-AQ group will decrease (from first expression to second and third expressions). The expression identification task was arranged after the presentation of the last face may drive attention and cause participants to pay disproportionate attention to the third expressions. Unfortunately, although this was anticipated and controlled for in the experiment, the expression identification task still drove the participant’s attention. This study showed that the amplitudes of P3 and LPC followed a decreasing trend from the first expressions to the second expressions, but then, increased with the appearance of the third expressions. This result is only partly in line with expectation. Whether the repeated stimuli led to information overload in this experiment could not be identified. However, as the ERP technology used in this study cannot be directly used to observe avoidance behavior, to better observe avoidance behavior of individuals with autistic traits, future studies could combine eye-tracking technology and increase the complexity and duration of presented stimuli.

## Conclusion

This study investigated the behavioral and neural mechanisms of the processing of repetitive facial expressions by individuals with autistic traits using ERP technology. Compared with the low-AQ group, individuals with autistic traits may focus an overly strong perception, attention, and evaluation of repetitive expressions, with a particular focus on negative expressions. This supports the intense world theory and helps to understand the neural processing of expressions in individuals with ASD.

## Supporting information

S1 File Data for every participant. (XLSX)

S1 Table Summary of statistical analysis results of ERP latency. (DOCX)

## References

[1] American Psychiatric Association. Diagnostic and statistical manual of mental disorders (DSM-5^®^) (5th ed.) Washington DC: American Psychiatric Pub.; 2013.

[2] Groot KD, Strien JV. Evidence for a broad autism phenotype. Advances in Neurodevelopmental Disorders. 2017;1(3):129–140. doi: 10.1007/s41252-017-0021-9

[3] Lai MC, Lombardo MV, Chakrabarti B, Baron-Cohen S. Subgrouping the autism "spectrum": reflections on DSM-5. PLoS biology. 2013;11(4):e1001544. doi: 10.1371/journal.pbio.1001544 . 23630456 PMC3635864

[4] Murray AL, Booth T, McKenzie K, Kuenssberg R, O’Donnell M. Are autistic traits measured equivalently in individuals with and without an autism spectrum disorder? An invariance analysis of the Autism Spectrum Quotient Short Form. Journal of autism and developmental disorders. 2014;44(1):55–64. doi: 10.1007/s10803-013-1851-6 23695223

[5] Baron-Cohen S, Wheelwright S, Skinner R, Martin J, Clubley E. The Autism-Spectrum Quotient (AQ): Evidence from Asperger Syndrome/High-Functioning Autism, Males and Females, Scientists and Mathematicians. Journal of Autism and Developmental Disorders. 2001;31(1):5–17. doi: 10.1023/a:1005653411471 11439754

[6] Poljac E, Poljac E, Wagemans J. Reduced accuracy and sensitivity in the perception of emotional facial expressions in individuals with high autism spectrum traits. Autism. 2013 Nov;17(6):668–680. doi: 10.1177/1362361312455703 . 22987888

[7] Halliday DWR, MacDonald SWS, Sherf SK, Tanaka JW. A Reciprocal Model of Face Recognition and Autistic Traits: Evidence from and Individual Differences Perspective. Plos One. 2014;9 (5):1–8. doi: 10.1371/journal.pone.0094013 24853862 PMC4031083

[8] Li X, Li Z, Xiang B, Meng J. Empathy for pain in Individuals with autistic traits influenced by attention cues: Evidence from an ERP study. Acta Psychologica Sinica 2020;52(3):294–306.

[9] Robinson EB, St Pourcain B, Anttila V, Kosmicki JA, Bulik-Sullivan B, Grove J, et al. Genetic risk for autism spectrum disorders and neuropsychiatric variation in the general population. Nature genetics. 2016 May;48(5):552–555. doi: 10.1038/ng.3529 . 26998691 PMC4986048

[10] Bralten J, van Hulzen KJ, Martens MB, Galesloot TE, Arias Vasquez A, Kiemeney LA, et al. Autism spectrum disorders and autistic traits share genetics and biology. Mol Psychiatry. 2018 May;23(5):1205–1212. doi: 10.1038/mp.2017.98 . 28507316 PMC5984081

[11] Takahashi J, Tamaki K, Yamawaki N. Autism spectrum, attachment styles, and social skills in university student. Creative Education. 2013;4(8):514–520. doi: 10.4236/ce.2013.48075

[12] Guan J, and Zhao X. Sub-Threshold Autistic Traits in Normal Population: Its Concept, Structure and Influencing Factors. Advances in Psychological Science. 2015;23(9):1599–1607. doi: 10.3724/SP.J.1042.2015.01599

[13] Robertson AE, Simmons DR. The relationship between sensory sensitivity and autistic traits in the general population. Journal of Autism and Developmental Disorders. 2013;43(4):775–784. doi: 10.1007/s10803-012-1608-7 22832890

[14] Baron-Cohen S. Mindblindness: An essay on autism and theory of mind. MIT Press. 1995.

[15] Grove R, Baillie A, Allison C, Baron-Cohen S, Hoekstra RA. The latent structure of cognitive and emotional empathy in individuals with autism, first-degree relatives and typical individuals. Molecular Autism. 2014;5(42):1–10. doi: 10.1186/2040-2392-5-42 25101164 PMC4123248

[16] Baron-Cohen S, Wheelwright S, Hill J, Raste Y, Plumb I. The ’Reading the Mind in the Eyes’ Test Revised Version: A Study with Normal Adults, and Adults with Asperger Syndrome or High-functioning Autism. Journal of Child Psychology and Psychiatry. 2001;42(2):241–51. doi: 10.1111/1469-7610.00715 11280420

[17] Lozier LM, Vanmeter JW, Marsh AA. Impairments in facial affect recognition associated with autism spectrum disorders: a meta-analysis. Development and psychopathology. 2014 Nov;26(4 Pt 1):933–945. doi: 10.1017/S0954579414000479 . 24915526

[18] Burt A, Hugrass L, Frith-Belvedere T, Crewther D. Insensitivity to Fearful Emotion for Early ERP Components in High Autistic Tendency Is Associated with Lower Magnocellular Efficiency. Frontiers in Human Neuroscience. 2017;11:1–12. doi: 10.3389/fnhum.2017.00001 29075185 PMC5643484

[19] Scambler DJ, Hepburn S, Rutherford MD, Wehner EA, Rogers SJ. Emotional responsivity in children with autism, children with other developmental disabilities, and children with typical development. Journal of autism and developmental disorders. 2007 Mar;37(3):553–563. doi: 10.1007/s10803-006-0186-y . 16933089

[20] Peterson C. Theory of mind understanding and empathic behavior in children with autism spectrum disorders. International Journal of Developmental Neuroscience. 2014;39:16–21. doi: 10.1016/j.ijdevneu.2014.05.002 24875777

[21] McKenzie K, Murray AL, Wilkinson A, Murray GC, Metcalfe D, O’Donnell M, et al. The relations between processing style, autistic-like traits, and emotion recognition in individuals with and without Autism Spectrum Disorder. Personality and Individual Differences. 2018; 120:1–6. doi: 10.1016/j.paid.2017.08.007

[22] Markram K, Markram H. The intense world theory a unifying theory of the neurobiology of autism. Frontiers in Human Neuroscience. 2010;4:1–29. 21191475 10.3389/fnhum.2010.00224 PMC3010743

[23] Millin R, Kolodny T, Flevaris AV, Kale AM, Schallmo MP, Gerdts J, et al. Reduced auditory cortical adaptation in autism spectrum disorder. Elife. 2018 Oct 26;7. doi: 10.7554/eLife.36493 . 30362457 PMC6203433

[24] Meng J, Li Z, Shen L. Altered neuronal habituation to hearing others’ pain in adults with autistic traits. Scientific reports. 2020 Sep 14;10(1):15019. doi: 10.1038/s41598-020-72217-x . 32929157 PMC7490706

[25] Kleinhans NM, Richards T, Greenson J, Dawson G, Aylward E. Altered Dynamics of the fMRI Response to Faces in Individuals with Autism. Journal of autism and developmental disorders. 2016 Jan;46(1):232–241. doi: 10.1007/s10803-015-2565-8 . 26340957 PMC4707097

[26] Green SA, Hernandez L, Lawrence KE, Liu J, Tsang T, Yeargin J, et al. Distinct Patterns of Neural Habituation and Generalization in Children and Adolescents With Autism With Low and High Sensory Overresponsivity. The American journal of psychiatry. 2019 Dec 1;176(12):1010–1020. doi: 10.1176/appi.ajp.2019.18121333 . 31230465 PMC6889004

[27] Baron-Cohen S, Ring HA, Bullmore ET, Wheelwright S, Ashwin C, Williams SCR. The amygdala theory of autism. Neuroscience and Biobehavioral Reviews. 2000;24:355–364. doi: 10.1016/s0149-7634(00)00011-7 10781695

[28] Bachevalier J, Loveland KA. The orbitofrontal-amygdala circuit and self-regulation of social-emotional behavior in autism. Neurosci Biobehav Rev. 2006;30(1):97–117. doi: 10.1016/j.neubiorev.2005.07.002 . 16157377

[29] Markram H, Rinaldi T, Markram K. The intense world syndrome—an alternative hypothesis for autism. Frontiers in Neuroscience. 2007;1(1):77–96. doi: 10.3389/neuro.01.1.1.006.2007 18982120 PMC2518049

[30] Monk CS, Weng S-J, Wiggins JL, Kurapati N, Louro HMC, Carrasco M, et al. Neural circuitry of emotional face processing in autism spectrum disorders. Journal of Psychiatry and Neuroscience. 2010;35(2):105–114. doi: 10.1503/jpn.090085 20184808 PMC2834792

[31] Dalton KM, Nacewicz BM, Johnstone T, Schaefer HS, Gernsbacher MA, Goldsmith HH, et al. Gaze fixation and the neural circuitry of face processing in autism. Nature Neuroscience. 2005;8(4):519–526. doi: 10.1038/nn1421 15750588 PMC4337787

[32] Lawson RP, Aylward J, White S, Rees G. A striking reduction of simple loudness adaptation in autism. Scientific reports. 2015 Nov 5;5:16157. doi: 10.1038/srep16157 . 26537694 PMC4633623

[33] Ghanouni P, Zwicker JG. Electrophysiological Responses to Emotional Facial Expressions in Individuals with Autism Spectrum Disorder: a Systematic Review. Review Journal of Autism and Developmental Disorders. 2018;5(3):208–226. doi: 10.1007/s40489-018-0134-8

[34] Luck SJ. An introduction to the Event-related potential technique. London: The MIT Press; 2005.

[35] Vlamings PHJM, Jonkman LM, van Daalen E, van der Gaag RJ, Kemner C. Basic Abnormalities in Visual Processing Affect Face Processing at an Early Age in Autism Spectrum Disorder. Biological Psychiatry. 2010;68(12):1107–1113. doi: 10.1016/j.biopsych.2010.06.024 20728876

[36] Allison T, Puce A, McCarthy G. Social perception from visual cues: role of the STS region. Trends in Cognitive Sciences. 2000;4(7): 267–278. doi: 10.1016/s1364-6613(00)01501-1 10859571

[37] Blau VC, Maurer U, Tottenham N, McCandliss BD. The face-specific N170 component is modulated by emotional facial expression. Behav Brain Funct. 2007 Jan 23;3(7):1–13. doi: 10.1186/1744-9081-3-7 . 17244356 PMC1794418

[38] Hajcak G, MacNamara A, Olvet DM. Event-Related Potentials, Emotion, and Emotion Regulation: An Integrative Review. Developmental Neuropsychology. 2010;35(2):129–55. doi: 10.1080/87565640903526504 20390599

[39] Li X, Zhang Y, Xiang B, Meng J. Empathy for face pain and its cognitive neural mechanism. Brain Science Advances. 2019;5(4):256–263. CNKI:SUN:LZSY.0.2019-04-003.

[40] Ito TA, Larsen JT, Smith NK, Cacioppo JT. Negative information weighs more heavily on the brain: The negativity bias in evaluative categorizations. Journal of Personality and Social Psychology. 1998;75(4). doi: 10.1037/0022-3514.75.4.887 9825526

[41] Lang PJ, Bradley MM. Emotion and the motivational brain. Biological Psychology 2010;84(3): 437–450. doi: 10.1016/j.biopsycho.2009.10.007 19879918 PMC3612949

[42] Schupp HT, Cuthbert BN, Bradley MM, Cacioppo JT, Ito T, Lang PJ. Affective picture processing: The late positive potential is modulated by motivational relevance. Psychophysiology. 2000;37(2):257–261. doi: 10.1111/1469-8986.3720257 10731776

[43] Ozonoff S, Miller JN. Teaching Theory of Mind: A New Approach to Social Skills Training for Individuals with Autism. Journal of Autism and Developmental Disorder. 1995;25(4):415–433. doi: 10.1007/BF02179376 7592252

[44] Lu M, Lei H, Su S, Ju S, Chen X. On the relationship between the abnormal sensory respondes and emotional / behavioral problems in children with autism spectrum disorders. Chinese Journal of Special Education. 2018 (4):60–65.

[45] Thye MD, Bednarz HM, Herringshaw AJ, Sartin EB, Kana RK. The impact of atypical sensory processing on social impairments in autism spectrum disorder. Dev Cogn Neurosci. 2018 Jan;29:151–167. doi: 10.1016/j.dcn.2017.04.010 . 28545994 PMC6987885

[46] Liu M. Screening Adults for Asperger Syndrome and High-Functioning Autism by Using the Autism-Spectrum Quotient (AQ) (Mandarin Version). Bulletin of Special Education. 2008;33(1):73–92.

[47] Meng J, Li Z, Shen L. Responses to others’ pain in adults with autistic traits: The influence of gender and stimuli modality. Plos One. 2017;12(3):1–12. doi: 10.1371/journal.pone.0174109 . 28319204 PMC5358845

[48] Meng J, Shen L, Li Z, Peng W. Top-down Effects on Empathy for Pain in Adults with Autistic Traits. Scientific reports. 2019;9(1). doi: 10.1038/s41598-019-44400-2 31142776 PMC6541648

[49] Bai L, Ma H, Huang YX, Luo YJ. The Development of Native Chinese Affective Picture System-A pretest in 46 College Students. Chinese Mental Health Journal. 2005;19(11):719–721. CNKI:SUN:ZXWS.0.2005-11-000.

[50] Iannetti GD, Hughes NP, Lee MC, Mouraux A. Determinants of Laser-Evoked EEG Responses: Pain Perception or Stimulus Saliency? Journal of Neurophysiology. 2008;100(2):815–828. doi: 10.1152/jn.00097.2008 18525021 PMC2525705

[51] Valentini E, Torta DME, Mouraux A, Iannetti GD. Dishabituation of Laser-evoked EEG Responses: Dissecting the Effect of Certain and Uncertain Changes in Stimulus Modality. Journal of Cognitive Neuroscience. 2011;23(10):2522–2537. doi: 10.1162/jocn.2011.21609 21265604

[52] Delorme A, Makeig SJ. EEGLAB: an open source toolbox for analysis of single-trial EEG dynamics including independent component analysis. Neurosci Methods 2004;134:9–21. doi: 10.1016/j.jneumeth.2003.10.009 15102499

[53] Jung TP, Makeig S, Westerfield M, Townsend J, Courchesne E, Sejnowski TJ. Analysis and Visualization of Single-Trial Event-Related Potentials. Human brain mapping. 2001;14:166–185. doi: 10.1002/hbm.1050 11559961 PMC6871967

[54] Batty M, Meaux E, Wittemeyer K, Rogé B, Taylor MJ. Early processing of emotional faces in children with autism: An event-related potential study. Journal of Experimental Child Psychology. 2011;109(4):430–444. doi: 10.1016/j.jecp.2011.02.001 21458825

[55] Wei P, Kang G, Ding J, Guo C. Monetary Incentives Modulate the Processing of Emotional Facial Expressions: An ERP Study. Acta Psychologica Sinica. 2014;46(4):437. doi: 10.3724/SP.J.1041.2014.00437

[56] Zhang D, Zhao T, Liu Y, Chen Y. Comparison of Facial Expressions and Body Expressions: An Event-related Potential Study. Acta Psychologica Sinica. 2015;47(8):963. doi: 10.3724/SP.J.1041.2015.00963

[57] Greenhouse SW, Geisser S. On methods in the analysis of profile data. Psychometrika 1959;24:95–112. doi: 10.1007/BF02289823

[58] Öhman A, Mineka S. Fears, phobias, and preparedness: Toward an evolved module of fear and fear learning. Psychological Review. 2001;108(3):483–522. doi: 10.1037/0033-295x.108.3.483 11488376

[59] Wallace GL, Case LK, Harms MB, Silvers JA, Kenworthy L, Martin A. Diminished sensitivity to sad facial expressions in high functioning autism spectrum disorders is associated with symptomatology and adaptive functioning. Journal of autism and developmental disorders. 2011 Nov;41(11):1475–1486. doi: 10.1007/s10803-010-1170-0 . 21347615 PMC3448486

[60] Whitaker LR, Simpson A, Roberson D. Brief Report: Is Impaired Classification of Subtle Facial Expressions in Children with Autism Spectrum Disorders Related to Atypical Emotion Category Boundaries? Journal of Autism and Developmental Disorders. 2017;47(8):2628–2634. doi: 10.1007/s10803-017-3174-5 28578469

[61] Taylor MJ, Batty M, Itier RJ. The faces of development: a review of early face processing over childhood. J Cogn Neurosci. 2004;16:1426–1442. doi: 10.1162/0898929042304732 15509388

[62] Kuefner D, de Heering A, Jacques C, Palmero-Soler E, Rossion B. Early Visually Evoked Electrophysiological Responses Over the Human Brain (P1, N170) Show Stable Patterns of Face-Sensitivity from 4 years to Adulthood. Front Hum Neurosci. 2010;3:1–22. doi: 10.3389/neur-o.09.067.2009 . 20130759 PMC2805434

[63] Akechi H, Senju A, Kikuchi Y, Tojo Y, Osanai H, Hasegawa T. The effect of gaze direction on the processing of facial expressions in children with autism spectrum disorder: an ERP study. Neuropsychologia. 2010;48(10):2841–2851. doi: 10.1016/j.neuropsychologia.2010.05.026 20546762

[64] Stavropoulos KKM, Viktorinova M, Naples A, Foss-Feig J, McPartland JC. Autistic traits modulate conscious and nonconscious face perception. Social neuroscience. 2016 Feb;13(1):40–51. doi: 10.1080/17470919.2016.1248788 . 27750521 PMC6194504

[65] Decety J, Yang C-Y, Cheng Y. Physicians down-regulate their pain empathy response: An event-related brain potential study. NeuroImage. 2010;50(4):1676–1682. doi: 10.1016/j.neuroimage.2010.01.025 20080194

[66] Fan Y-T, Chen C, Chen S-C, Decety J, Cheng Y. Empathic arousal and social understanding in individuals with autism: evidence from fMRI and ERP measurements. Social cognitive and affective neuroscience. 2014;9(8):1203–1213. doi: 10.1093/scan/nst101 23929944 PMC4127026

[67] Ghosn F, Perea M, Castello J, Vazquez MA, Yanez N, Marcos I, et al. Attentional Patterns to Emotional Faces Versus Scenes in Children with Autism Spectrum Disorders. Journal of autism and developmental disorders. 2019 Apr;49(4):1484–1492. doi: 10.1007/s10803-018-3847-8 . 30536217

[68] Leutgeb V, Schäfer A, Schienle A. An event-related potential study on exposure therapy for patients suffering from spider phobia. Biological psychology. 2009;82(3):293–300. doi: 10.1016/j.biopsycho.2009.09.003 19751797

[69] Kleinhans NM, Richards T, Weaver K, Johnson LC, Greenson J, Dawson G, et al. Association between amygdala response to emotional faces and social anxiety in autism spectrum disorders. Neuropsychologia. 2010 Oct;48(12):3665–70. doi: 10.1016/j.neuropsychologia.2010.07.022 . 20655320 PMC3426451

[70] Leekam SR, Nieto C, Libby SJ, Wing L, Gould J. Describing the sensory abnormalities of children and adults with autism. Journal of autism and developmental disorders. 2007 May;37(5):894–910. doi: 10.1007/s10803-006-0218-7 . 17016677

