# Supplementary material for: Processing of expressions by individuals with autistic traits: Empathy deficit or sensory hyper-reactivity?
Source: PLoS One. 2021 Jul 9;16(7):e0254207. doi: 10.1371/journal.pone.0254207 (PMC8270190; doi:10.1371/journal.pone.0254207)
Supplement: S1 Table — (DOCX) [file pone.0254207.s002.docx]

**S1 Table. Summary of statistical analysis results of ERP data (latency).**

|  | P1 | | | N170 | | | P3 | | |
| --- | --- | --- | --- | --- | --- | --- | --- | --- | --- |
|  | *F* | *p* | η^2^_p_ | *F* | *p* | η^2^_p_ | *F* | *p* | η^2^_p_ |
| Expression | 0.36 | 0.700 | 0.01 | 1.19 | 0.313 | 0.04 | 0.80 | 0.458 | 0.03 |
| Sequence | 2.88 | 0.064 | 0.09 | 0.72 | 0.494 | 0.02 | **5.80** | **0.005** | **0.17** |
| Group | 0.03 | 0.875 | <0.001 | 3.01 | 0.088 | 0.05 | <0.01 | 0.948 | <0.001 |
| Expression ×Group | 0.10 | 0.908 | <0.01 | 0.01 | 0.985 | <0.011 | 1.45 | 0.246 | 0.03 |
| Sequence ×Group | 0.70 | 0.503 | 0.02 | 0.58 | 0.563 | 0.02 | 0.58 | 0.563 | 0.02 |
| Expression × Sequence | 0.88 | 0.485 | 0.06 | **2.93** | **0.029** | **0.18** | 1.44 | 0.246 | 0.05 |
| Expression × Sequence×Group | 1.05 | 0.392 | 0.07 | 0.96 | 0.436 | 0.07 | 0.20 | 0.936 | 0.01 |

Note: df:(1,58), The significant comparisons (*p* ﹤0.05)were shown in boldface.
